# Clinical target segmentation using a novel deep neural network: double attention Res-U-Net

**DOI:** 10.1038/s41598-022-10429-z

**Published:** 2022-04-25

**Authors:** Vahid Ashkani Chenarlogh, Ali Shabanzadeh, Mostafa Ghelich Oghli, Nasim Sirjani, Sahar Farzin Moghadam, Ardavan Akhavan, Hossein Arabi, Isaac Shiri, Zahra Shabanzadeh, Morteza Sanei Taheri, Mohammad Kazem Tarzamni

**Affiliations:** 1 Research and Development Department, Med Fanavaran Plus Co., Karaj, Iran; 2 Department of Electrical and Computer Engineering, National Center for Audiology, Western University, London, Canada; 3 Department of Cardiovascular Sciences, KU Leuven, Leuven, Belgium; 4 Division of Nuclear Medicine and Molecular Imaging, Geneva University Hospital, 1211 Geneva 4, Switzerland; 5 School of Medicine, Shahid Beheshti University of Medical Sciences, Tehran, Iran; 6 Department of Radiology, Shohada-e-Tajrish Hospital, Shahid Beheshti University of Medical Sciences, Tehran, Iran; 7 Department of Radiology, Imam Reza Hospital, Tabriz University of Medical Sciences, Tabriz, Iran

**Keywords:** Biomedical engineering, Computational science

## Abstract

We introduced Double Attention Res-U-Net architecture to address medical image segmentation problem in different medical imaging system. Accurate medical image segmentation suffers from some challenges including, difficulty of different interest object modeling, presence of noise, and signal dropout throughout the measurement. The base line image segmentation approaches are not sufficient for complex target segmentation throughout the various medical image types. To overcome the issues, a novel U-Net-based model proposed that consists of two consecutive networks with five and four encoding and decoding levels respectively. In each of networks, there are four residual blocks between the encoder-decoder path and skip connections that help the networks to tackle the vanishing gradient problem, followed by the multi-scale attention gates to generate richer contextual information. To evaluate our architecture, we investigated three distinct data-sets, (i.e., CVC-ClinicDB dataset, Multi-site MRI dataset, and a collected ultrasound dataset). The proposed algorithm achieved Dice and Jaccard coefficients of 95.79%, 91.62%, respectively for CRL, and 93.84% and 89.08% for fetal foot segmentation. Moreover, the proposed model outperformed the state-of-the-art U-Net based model on the external CVC-ClinicDB, and multi-site MRI datasets with Dice and Jaccard coefficients of 83%, 75.31% for CVC-ClinicDB, and 92.07% and 87.14% for multi-site MRI dataset, respectively.

## Introduction

Accurate medical image segmentation in clinics plays a pivotal role in precise and accurate diagnosis; however, automated segmentation tasks face certain challenges in clinical practice^1^. Automated medical image segmentation has been considered to help clinicians to achieve a more accurate diagnosis. To this end, image segmentation algorithms have focused on extracting various feature maps associated with the target structure in order to predict/identify the target class, anatomy, or structure from the input images. Recently, owing to the substantial progress in digital medical imaging systems, more attention has been paid towards applying complex image processing algorithms to address medical image analysis task and automated medical image segmentation^2^. The aim of medical image segmentation is to help clinicians by concentrating on a particular region of interest and extracting detailed information for diagnosis. Traditional image segmentation algorithms mostly relied on handcrafted features like texture, color, and shapes^3–8^. Although traditional machine learning approaches have been successful for image segmentation to a certain extent, these solutions do not perform well in complex problems and challenging tasks^7,8^. In addition, the comprehensive modeling of complicated phenomena is another limitation of traditional machine learning approaches. Moreover, traditional medical image segmentation methods cannot result in reliable segmentation performance when face with different types of medical images. Thus, various types of medical images including ultrasound images, colonoscopy images, and MRI images have been experimented in this paper to show the robustness of the proposed structure in comparison with other approaches.

Segmentation of various targets in different medical images has been studied in this manuscript containing fetal organ segmentation in ultrasound images, colon tumors in colonoscopy images and prostate in abdominal MRI images. Automatic fetal anatomies segmentation using ultrasound images has been considered in some researches^9–13^. Jardim et al.^12^, has mentioned one possible reason for this point which is the low resolution quality of ultrasound images. This deficiency is mainly due to the high ratio of noise, different data collecting approaches, differences in the gestational ages due to the shape of the fetal body, and large intra class due to the dynamic body parts. Morphological operators are the initial methods for automatic segmentation of fetal biometry in ultrasound images^11,13,14^. Edge detection, edge linking, Hough transform are the main series of steps in morphological operators, to provide head and femur segmentation for fetal biometry analysis purpose. Chalana et al.^9^, and Chervenak et al.^10^ studied methods for fetal head and abdomen segmentation in ultrasound images, respectively. Jardim et al.^12^ proposed an approaches for fetal segmentation in ultrasound images by the evolution of a parametric deformable shape. Moreover, in some researches, colonoscopy and MRI data have been used for segmentation task. A parallel reverse attention network has proposed for polyp segmentation in colonoscopy images. In^15^, the authors have used parallel decoder in order to summation the high level features which combination of these features generated a global map for other components in the proposed strategy. Ghose et al.^16^ have used a supervised learning framework of random forest algorithm to achieve a probabilistic of prostate voxel segmentation in MRI images. Wavelet multi-scale domain for MRI prostate segmentation and discriminate noise has been studied by Flores-Tapia et al.^17^.

Recently, the use of deep learning-based architectures has remarkably increased due to their capability of extracting features automatically from the input data. Therefore, these approaches enable us to overcome the limitations of traditional algorithms. These methods have shown promising results for many tasks such as image classification^18^ biomedical image segmentation^19^, and^20^. Zhou et al.^20^ have used deep convolutional neural networks for medical image segmentation. In their study, the authors focused on maintaining the spatial dimension of feature maps in different layers using atrous convolutions. Fully convolutional neural networks have been applied to colonoscopy images for polyp segmentation in^21–23^. Prostate segmentation in MRI images using convolutional neural network has been studied by Karimi et al.^24^. The proposed neural network in this paper segmented the prostate key-points by calculating the center and the parameters of the prostate shape.

Among various deep learning-based solutions for medical image segmentation, U-Net architecture has attracted the most attention in research settings. Ronnerberger et al.^4^ proposed a U-Net model which included two main modules: an encoder module and a decoder module. These modules were connected to each other via skip connections. Various blocks of neural networks in the encoder modules were employed to extract a large number of feature maps from the input data. In the decoder modules, transposed convolution has been exploited to produce segmentation maps from the localized region. Various promotions of U-Net architectures, mostly differ in their skipping connections, have been proposed^25–28^. Seo et al.^29^ has introduced a Modified U-Net (mU-Net) for liver and liver tumor segmentation from CT images. They have applied a residual module with deconvolution and activation operations through the skip connection of the U-Net model to address the problem of low resolution information of features in U-Net structure. Owing to the promising results obtained from the U-Net structure, this architecture has been used in the analysis of various types of medical images like MRI data for the segmentation of cartilage and meniscus^30^, and CT data to segment lung^31^. SE-U-Net that is a U-Net network augmented by the dilation kernel to segment the polyp in colonoscopy images has proposed by Guo et al.^32^. In^33^, a modified encoder-decoder with several integrated sequential depth dilated inception blocks based on deep learning has proposed to overcome limitations of traditional approaches by aggregating features from different receptive area of dilated convolutions for polyp segmentation from colonoscopy images. Cascade dense U-Net for prostate segmentation in MRI images has studied by Li et al.^34^. In this method, at first, a dense U-Net model has used for initial segmentation, and these segmentation results used as prior knowledge for another dense U-Net to get more accurate segmentation result. Moradi et al.^35^ proposed Multi-Feature Pyramid U-Net (MFP U-Net) model for left ventricle segmentation. They equalized the depth of all feature maps within the decoder path in order to increase segmentation accuracy. Automated concentration on different regions of interest and/or targets through the use of Attention Gates (AGs), known as Attention U-Net model, has been proposed by Oktay et al.^26^. Generating different scales of context information without any information loss is one of the dilated advantages of this model, which has been proposed in^36^.

Although there are various U-Net-based architectures for medical image segmentation, there is no study and dedicated architecture specially for various clinical targets segmentation in different image types. Therefore, in this study ultrasound imaging systems (for Crown Rump Length (CRL) and fetal foot segmentation), colonoscopy images for polyp segmentation, and MRI images for prostate segmentation has been used. We proposed a novel Double Attention Res-U-Net architecture that experimented using three distinct datasets, in order to show the robustness of the proposed model in using different types of medical data. To this end, the proposed model used for CRL and fetal foot segmentation in ultrasound images (During pregnancy, the measurement of CRL and fetal foot is critical for calculating the gestational age and fetal weight. This gestational age allows doctors to estimate the potential due date), polyp segmentation in colonoscopy imaging system as well as prostate segmentation throughout the MRI images. Therefore, the automatic medical image segmentation for the automation of measurements using the proposed method has the potential of:

1. Improving clinician target segmentation task in various type of medical images.
2. Improving accuracy and consistency of measurements in various type of medical images.
3. Accurate segmentation in face of the challenging targets.

In summary, the proposed approach in this paper is designed to doing automatic task in order to detect and segment the CRL, fetal foot, polyp and prostate segmentation. The raw input images are fed to the system and then the measurement to be performed. Extensive experiments result that, on average, the output performance by our system is more close to the annotation accuracy made by experts for the measurements mentioned above and has stability in face of challenging segmentation targets as well as various type of medical images.

The rest of the paper is organized as follows: We described the double attention Res-U-Net in “The proposed architecture” section. Experiments and results are presented in “Results” section. The validity of the results are discussed in the “Discussion” section and finally, the statement of the paper is summarized in the “Conclusion” sections.

## The proposed architecture

**Figure 1:**
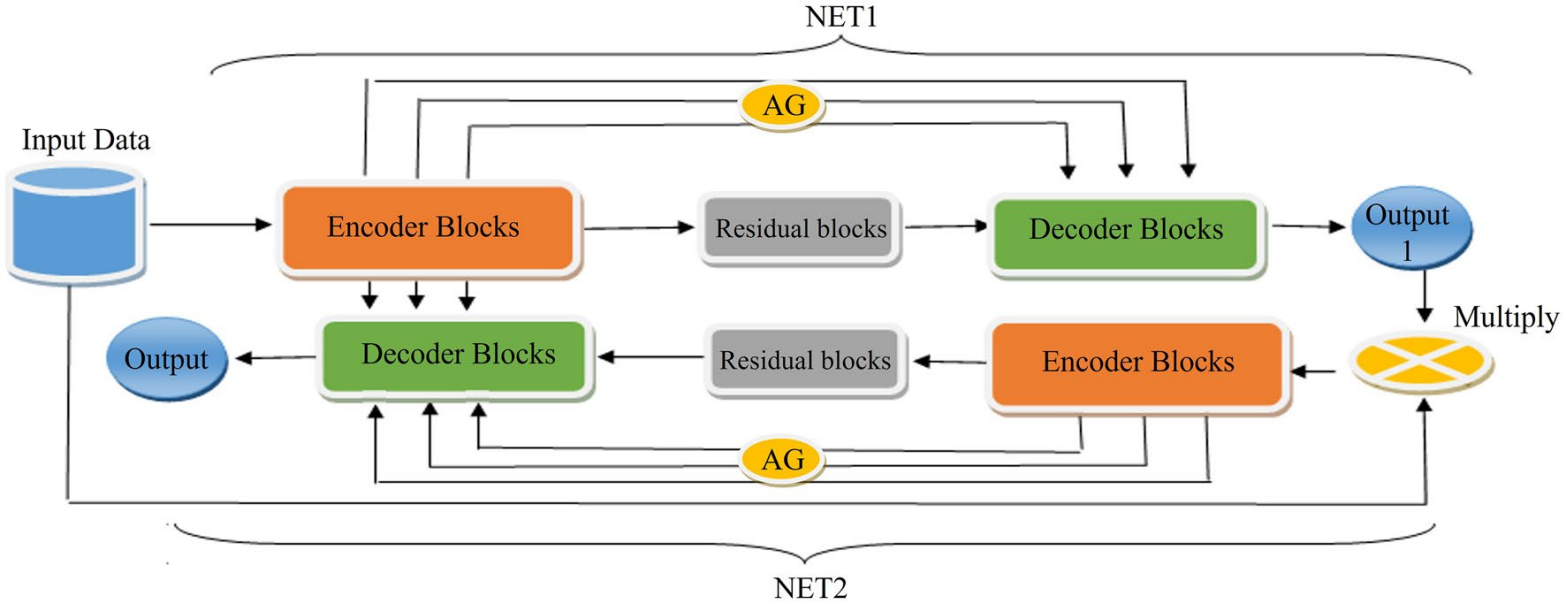
Overview of the proposed double attention Res-U-Net architecture.

**Figure 2:**
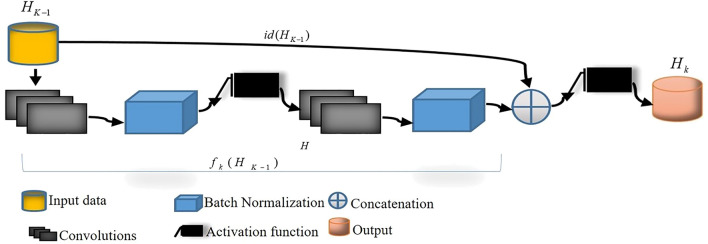
Overview of the proposed residual block.

**Figure 3:**
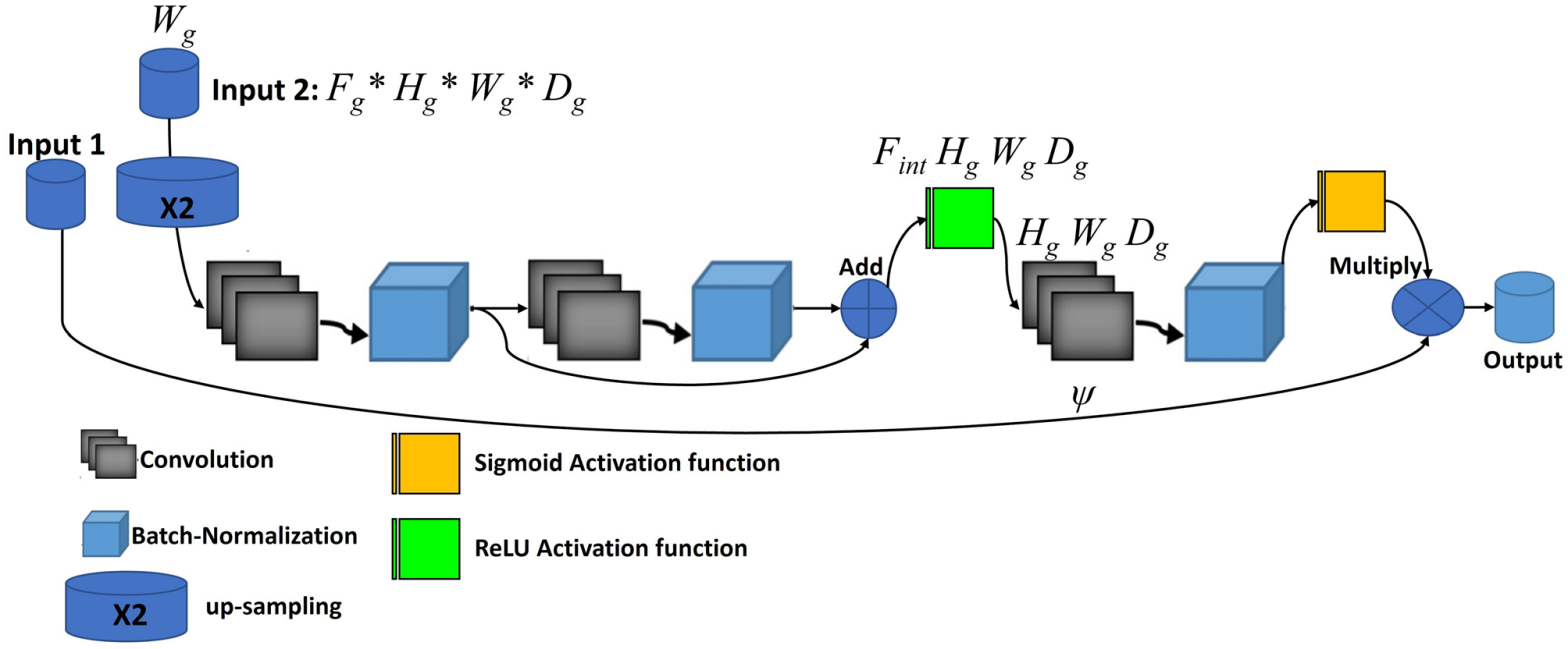
Overview of the proposed attention gate architecture.

Figure 1 illustrates an overview of the proposed architecture wherein two subsequent networks (i.e., NET1 and NET2) are used. Each of these networks consists of four main encoder blocks, five decoder blocks, a residual block, and AGs. We have used a residual block between the encoder and decoder paths in both networks (NET1 and NET2), which is shown in Fig. 2. The input of NET2 is an element-wise multiplication of the output of NET1 with the input data of the NET1. An AG has been used within the skip connection of both networks. It enables the network to replace less effective feature maps with the key features for the given task. In the proposed structure, AGs are used in different scales including different semantic features that stack-up the information from different scales, which improve the grid-resolution of the target signal and achieve better output. The AGs structure has illustrated in Fig. 3. This multi-scale strategy encourages the model to extract/generate richer contextual information at different resolutions. It also greatly increases the effectiveness of the feature maps. It should be noted that the abovementioned settings are common for both NET1 and NET2.

In recent segmentation approaches^37–39^, object localization models have been used to divided the task into separate localization and succedent segmentation steps in order to achieve sufficient segmentation accuracy. AG is a standard convolutional neural network that is integrated into the proposed model to enhance computational/performance efficiency with minimal computational overhead. The proposed AG module would improve the segmentation performance across the different types of medical images through increasing the model sensitivity. Features of irrelevant background zone without attention to crop a ROI between networks have been used using AGs blocks, that show the noticeable pros in comparison with the base localization models. Attention coefficients, $\left( {\alpha_{i} } \right)$ ∈ [0, 1], highlight the salient parts of image and preserve the activations of the specific task with omitting feature responses. Feature-maps of input multiplied by attention coefficients that results in the output of AGs: $\hat x_{i,c}^l = x_{i,c}^l \cdot \alpha _i^l$. A single scalar attention value for each pixel vector is computed: $x_{i}^{l} \in R^{{F_{l} }}$ where $F_{l}$ refer to the number of feature-maps in layer “l”. We have used multi-dimensional attention coefficients to learn multiple semantic classes. Thus, each AG learns to concentrate on a different subset of target structures through a U-Net model. We have used additive attention^40^ that is computationally expensive, but achieves higher accuracy^41^. Additive attention is formulated as follows:

1 $$q_{att}^{l} = \psi^{T} (\sigma_{1} (W_{x}^{T} x_{i}^{l} + W_{g}^{T} g_{i} + b_{g} )) + b_{\psi }$$

2 $$\alpha_{i}^{l} = \sigma_{2} (q_{att}^{l} (x_{i}^{l} ,g_{i} ;\theta_{att} ))$$

Here, the feature maps ($n_{x}$) from the encoder layer are represented by x. The corresponding $n_{g}$ feature maps from decoder path, that are typically concatenated with x in the skip connection, are represented by g. $\sigma_{2} (x_{i} ,c) = \frac{1}{{1 + \exp ( - x_{i,c} )}}$ indicates sigmoid activation function. $W_{x}$ and $W_{g}$ depict 1 × 1 ×  1 convolution kernel to extract $n_{x}$ features. $\psi$ is a 1 × 1 × 1 convolution kernel, which results in 1 feature map. $\sigma_{1}$ is a ReLU activation function and the “b” vectors are bias terms. AG is characterized by a set of parameters $\theta_{att}$ including linear transformations $W_{x} \in R^{{F_{l} *F_{{\text{int}}} }} ,W_{g} \in R^{{F_{g} *F_{{\text{int}}} }} ,\psi \in R^{{F_{{\text{int}}} *1}}$ and bias terms $b_{\psi } \in R,b_{g} \in R^{{F_{{\text{int}}} }}$. In our linear transformation, at first, in order to omit the one block in decoding path, the input from decoder paths to AG (black arrow) has up-sampled by 2, and then 1 × 1 × 1 convolutions have been applied on the input tensors. Omitting the block in decoder path help the network to decrease some computation process and therefore decrease the training parameters. In^42^, authors referred to the linear transformation by concatenating the attention vector, where the merged features including $x^{l}$ and g are mapped to a $R^{{F_{{\text{int}}} }}$ dimensional intermediate features in a linear manner. Sequential use of softmax activation function leads to sparser output^43,44^. To this reason, we have used a sigmoid activation function in the proposed algorithm which yields better training convergence for the parameters of the AGs. Sampling based update have been used in hard-attention^45^ methods, but in the proposed structure, standard back-propagation strategy performed to train the AGs parameters.

The proposed AGs are applied to the proposed double network with two standard U-Net architecture which force the networks to concentrate on salient features through the skip connections (see Fig. 1). AGs have used right before the concatenation operation with two main works during the forward and backward directions including merge the relevant activations and filter the neuron activations. Gradients descent from background has performed throughout the backward pass, where the AGs parameters updated based on spatial relevant regions. In order to decrease the number of trainable parameters and computational complexity of AGs, the linear transformations with 1 × 1 × 1 convolutions performed and then the feature-maps are down-sampled to the resolution of gating signal. In Eqs. (1) and (2), $W_{x}$ and represent 1 × 1 × 1 convolution operations that generate $n_{x}$ features, wherein $\psi$ is a 1 × 1 ×  1 convolution kernel intended to output a single feature map. This indicates that there is a 1 × 1 convolution kernel with 1 convolutional filter to extract one feature map. The 1 × 1 filter is often called a feature map pooling layer which provides efficient feature maps from the input data to the AGs that come from encoder path. Thus, with 1 × 1 ×  1 kernels in each skip-connection there is a feature map that the width and height of feature map remain unchanged regarding the size of input feature map of the different blocks of the encoder path. Using the AGs within the skip connection between the encoder and decoder path, ‘gating’ the incoming feature maps from the encoder path. Thus, through generating one feature map from multiple incoming feature maps from encode path, the number of trainable parameters and computational complexity decreased owing to this gating mechanism. We have used the strategy of^46^ that know as deep-supervision to semantically discriminative the intermediate feature-maps from each image scale. This strategy helps the different scales of attention units to influence the content of image foreground which can exclude reconstructed dense predictions from small subsets throughout the skip connections that leads to represent the input data in a low dimensional space. For more information, the details architecture along with tuned parameters of encoder, decoder, and residual blocks are described below. It is noticeable that the optimum layers and hyper-parameters were achieved through a grid search scheme.

### The encoder module

This module is comprised of three main layers including a 2D convolutional layer, a batch normalization layer^47^, and a Leaky Rectified Linear Units (Leaky ReLU) with an activation function along with 0.2 negative slope coefficient. We initialized these convolutional layers randomly by normal distribution with a standard deviation of 0.02. We have used a convolutional kernel of 5 × 5 which is padded and swept by 2 × 2 stride. Generally, we have used five encoder blocks with the same settings (hyper-parameters). In order to extract various feature maps in different blocks, 20, 40, 80, 160, and 320 convolutional filters have been used within the five layers, respectively.

### The decoder module

In the proposed architecture, we have used four 2D transposed convolution layers that are padded and initialized randomly using a normal distribution with a standard deviation of 0.02. A 5 × 5 kernel with 2 × 2 stride sweep over the inputs in all decoder layers. We have used 160, 80, 40, and 20 filters in the four deconvolution layers, respectively. Each deconvolution layer was followed by a batch normalization layer and a dropout layer with a probability of 30% to avoid overfitting during the training. Finally, Rectified Linear Units (ReLU)^48^ was employed as activation function after concatenating each batch normalization layer with the corresponding skip connection feature maps from the first encoder. While in the second decoder, skip connections were employed to connect both of the encoders.

### The residual blocks

The encoded features were processed with four Residual layers that consisted of skip connections and were followed by a series of decoder blocks to account for the size of the output image. The residual blocks are comprised of two padded convolutional layers where the input to the block is concatenated to the output of the block. The residual block uses two convolution layers with 320 filters, 5 × 5 kernel size, 1 × 1 stride, without any ReLU activation function after the second block. Residual blocks help the network to tackle the vanishing gradient problem using identity mapping. The merit of the proposed residual block in comparison to the typical residual blocks is that this module employes batch normalization layers after convolutional layers to accelerate the training task. In Fig. 2, the residual block is defined as $H_{k} = F(H_{K - 1} ,W_{k} ) + H_{k - 1}$. Here, $H_{k - 1}$ is the input to the residual block, $H_{k}$ is the output of the block and $W_{k}$ are the trainable weights for the mapping of function F.

Given input data $X_{in}$, the operations of the encoder block, residual block, and decoder block of the NET1 have been indicated by E, R, D, respectively, in Eq. (3). AG refers to attention gate in this formula that is concatenated with the features of decoder path. $X_{out1}$ is the outcome of NET1. In Eq. (4), $X_{out1}$ * $X_{in}$ denote the multiplication of the input image with the output salient of NET1. E, R, and D represent the encoder block, residual block, and decoder block of the NET2. AG refers to the attention gate of the NET1 and NET2 in Eq. (4). The general proposed structure in this research can be formulized as follow:

3 $$X_{out1} = \sum \left[ {X_{in} \to (E_{1} ) + R_{1} \to Concat(AG_{1} ,D_{1} )} \right]$$

4 $$X_{out2} = \sum \left[ {(X_{out1} *X_{in} ) \to (E_{2} ) + R_{2} \to Concat(AG_{1} ,AG_{2} ,D_{2} )} \right]$$

**Figure 4:**
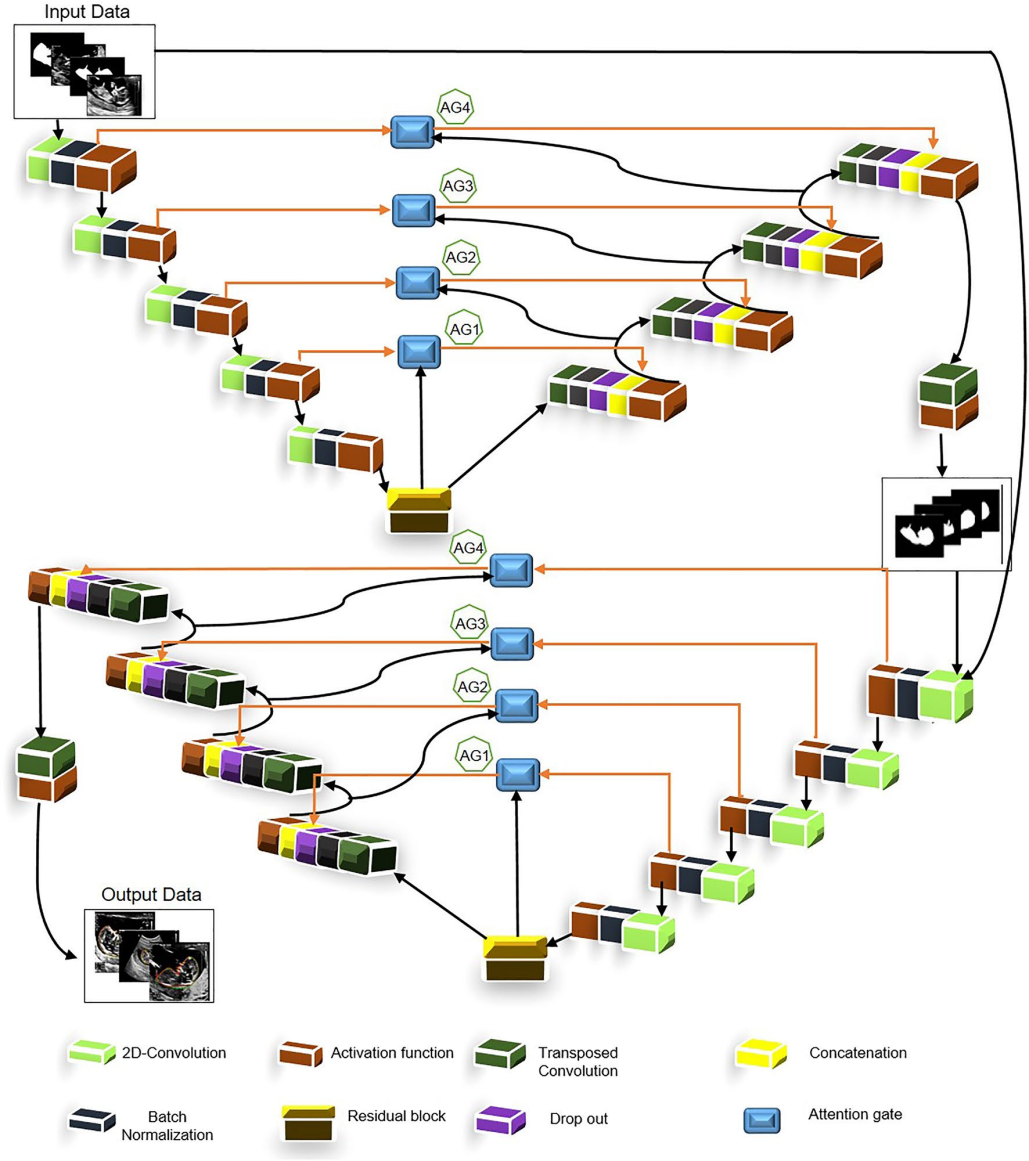
Complete structure of the proposed model.

Finally, sigmoid function has been used through a 2D transposed convolution layer to generate the corresponding mask. This layer is padded and initialized using random normal with 0.02 as standard deviation. Convolution kernel size was 5 × 5 with a stride of 2 × 2. The optimization method was Adam with a learning rate of 0.0001. Figure 4 illustrated the proposed model’s architecture in details. To make the figure uncomplicated, we have used pointer to show the connection between AG1 to AG4, and means that point from first network concatenated with the corresponding point in the second network. For instance, AG1 in first network concatenated with AG1 point in the second network.

It is worthy to note that in order to achieve high accuracy, the proposed model relies on higher number of trainable parameters. Higher number of trainable parameters improved the efficiency of the feature extraction and distinction of the target structure from the background in such a way that the total amount of computation across and within different layers did not increase. This is due to the fact that higher number of parameters bypassed the complex computations or replaced complex compactions with simple ones using larger number of parameters. As a result, the total computational complexity or processing time reduced with the proposed architecture due to the optimization process and well structure of the model in comparison with other models.

## Experiments

### Dataset

We conducted the experiments on three datasets with different image types.

- A clinical dataset of ultrasound images for the task of CRL and fetal foot segmentation.
- CVC-ClinicDB dataset^49^, for polyp segmentation.
- Multi-site MRI dataset^50^, for prostate segmentation

#### Collected dataset

**Figure 5:**
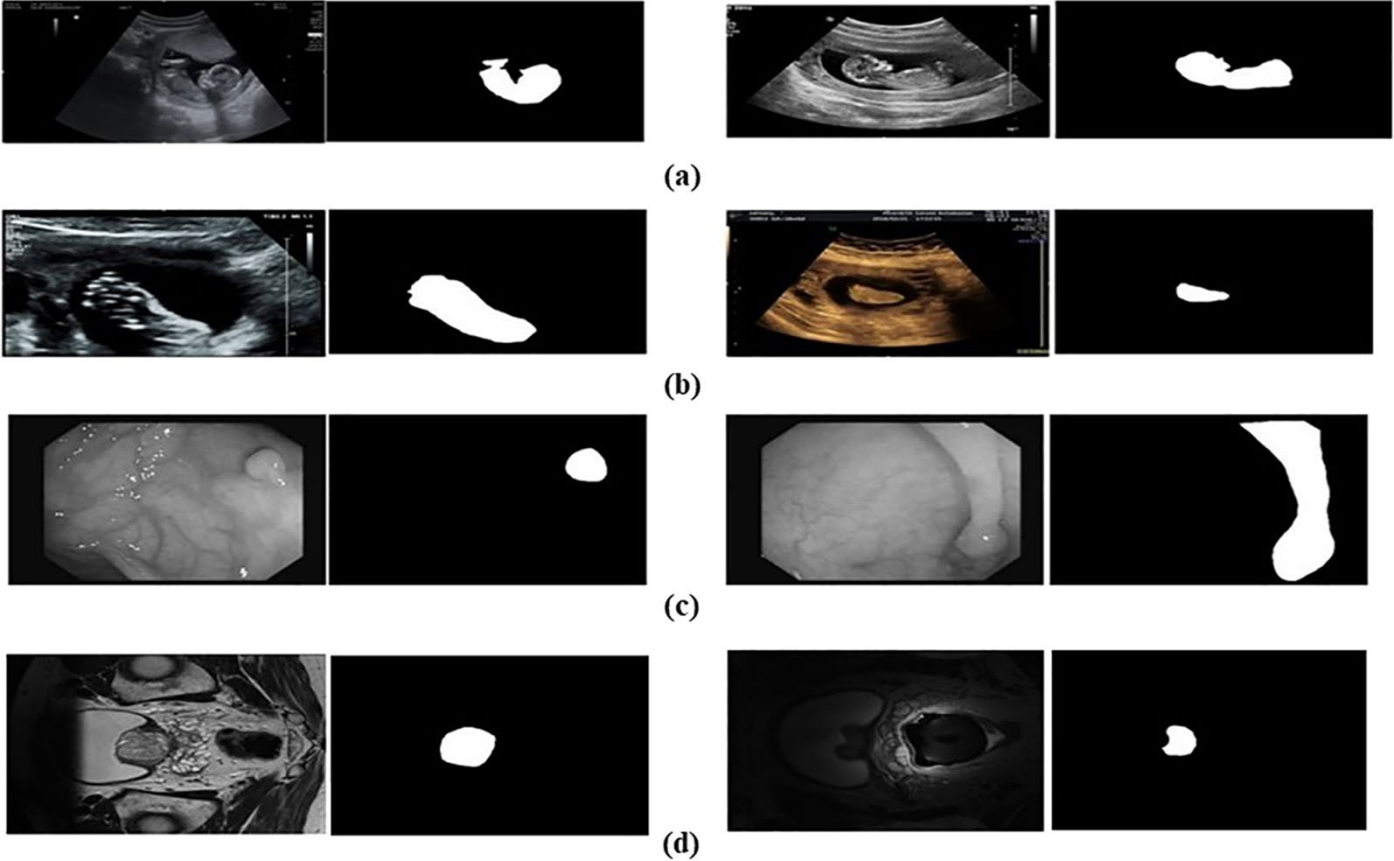
Samples of the CRL Foot-MFP, CVC-ClinicDB, and multi-site MRI datasets together with their corresponding annotation of the target structures. CRL (**a**), Fetal Foot (**b**), CVC-ClinicDB (**c**), Multi-site MRI (**d**).

This dataset (referred to as CRL Foot-MFP) consisted of 525 samples for CRL and 1119 images for fetal foot class that has used to evaluate the proposed method in this paper. The original size of the collected images was 1024 × 768 pixels acquired from SIMUT Luna Pro ultrasound scanners. Figure 5a,b show some samples of CRL and fetal foot. The right images are the equivalent masks of fetal foot and CRL of left images.

#### CVC-ClinicDB dataset

In addition, we conducted experiments on a standard benchmark dataset known as CVC-ClincDB. This dataset consisted of 612 Polyp images with the size of 384 × 288 pixels. Some samples of the CVC-ClinicDB dataset as well as equivalent masks are shown in Fig. 5c. The right images are the equivalent masks of polyp images.

#### Multi-site MRI dataset

This dataset comprised of multi-site MRI data (T2-weighted MRI data) for prostate segmentation. This dataset collected out of three public sources. We randomly selected the samples of three sites D, E, F from this dataset to compare with other researches. The detail information of each site including number of samples, image resolution, and imaging protocols are summarized in the Table 1.

**Table 1:** The details of sample number and imaging protocols in the multi-site MRI dataset.

Dataset	Institution	Case num	Field strength (T)	Resolution (mm)	Endorectal coil	Manufactor
Site D	UCL	13	1.5 and 3	0.325–0.625/3–3.6	No	Siemens
Site E	BIDMC	12	3	0.25/2.2–3	Endorectal	GE
Site F	HK	12	1.5	0.625/3.6	Endorectal	Siemens

### Pre-processing

The proposed model was independently trained using three datasets. We have randomly selected 20% of the datasets to evaluate the model in test phase. In addition, we have used 80% of each dataset to train the model independently from each dataset. Ultrasound images in the CRL Foot-MFP and multi-site MRI datasets dataset are resized to 472 × 320 pixels resolution. For the CVC-ClinicDB dataset, we have used the original size of the images. In multi-site MRI dataset, we have omitted the images that does not include masks. The entire input images were converted to gray scale and normalized by their standard deviation prior to the training of the model. Normalizing formula is determined as below where $x_{i}$ and $\overline{{x_{i} }}$ are the element and normalized element, respectively and *s* is the standard deviation of *x*.

5 $$\mathop x\limits^{ - } = \frac{{x_{i} - mean(x)}}{s}$$

### Evaluation metrics

First, in order to compare the estimated volumes of the target structures, we used the Dice Similarity Coefficient (DSC). Further, we assessed the segmentation performance based on the Jaccard Similarity Coefficient (JSC), and Hassdorff Distance (HD) between the ground truth contours (defined manually) and the predicted one. The DSC indices were calculated using Eq. (6), where ${\mathrm{A}}_{\mathrm{M}}$ indicates ground truth contours and ${\mathrm{A}}_{\mathrm{A}}$ is the predicted contours by the model. Jaccard similarity coefficients were calculated using Eq. (7).

6 $$\mathrm{DSC}=\frac{2\left({\mathrm{A}}_{\mathrm{A}}\cap {\mathrm{A}}_{\mathrm{M}}\right)}{{\mathrm{A}}_{\mathrm{A}}+{\mathrm{A}}_{\mathrm{M}}}$$

7 $$\mathrm{J}\left(\mathrm{A},\mathrm{ B}\right)=\frac{\left|\mathrm{A}\cap \mathrm{B}\right|}{\left|\mathrm{A}\cup \mathrm{B}\right|}=\frac{\left|\mathrm{A}\cap \mathrm{B}\right|}{\left|\mathrm{A}\right|+\left|\mathrm{B}\right|-\left|\mathrm{A}\cap \mathrm{B}\right|}$$

In addition, in order to measure the maximum distance of the predicted contour to the nearest point in the reference contours, we have calculated HD (Eq. 8). “A” and “B” denote the two contours, where d (a, b) indicates Euclidean distance. In this paper, we used the Dice coefficient loss function^51,52^.

8 $$HD = \max (\mathop {\max }\limits_{a \in A} (\mathop {\min }\limits_{b \in B} d(a,b)),\mathop {\max }\limits_{b \in B} (\mathop {\min }\limits_{a \in A} d(a,b)))$$

## Results

In this section, we provide the results of the proposed architecture in comparison with state-of-the-art U-Net-based architectures on the CRL Foot-MFP, CVC-ClinicDB, and multi-site MRI datasets. We compared the proposed model with U-Net^4^, dilated U-Net^36^, attention U-Net^26^, R2 U-Net^25^, and MFP-U-Net^35^ architectures, considered as the state-of-the-art deep-learning algorithms in medical image segmentation.

### Experiments on CRL Foot-MFP dataset

The results of the experiments on CRL Foot-MFP dataset for CRL and fetal foot segmentation are summarized in Table 2. In this table, the mean accuracy of Dice and Jaccard along with the standard deviation values for each class were expressed. For the CRL segmentation, the proposed model achieved Dice and Jaccard coefficients of 95.79% and 91.62%, respectively, outperforming other U-Net-based models. From Table 2, we can also observe that for CRL measurement, the other five U-Net-based models have exhibited very competitive accuracy rates. In this table, we also calculated HD for 105 sample tests of CRL, and higher values of HD indicated that the two contours do not match closely. In this regard, the proposed model achieved an HD of 35.9 mm that was very close to the dilated U-Net model with an HD of 35.7 mm. R2U-Net exhibited the worst result with an HD of 39.19 mm.

**Table 2:** Comparison of test results for CRL and Foot segmentation from CRL and Foot-MFP dataset Numbers format (mean value ± standard deviation).

CRL						Foot					
Models	DSC	JSC	HD	DSC P-value	JSC P-value	Models	DSC	JSC	HD	DSC P-value	JSC P-value
Proposed model	**95.79** ± 0.01	**91.62** ± 0.01	35.90	8.80 × ${10}^{-59}$	2.93 × ${10}^{-58}$	Proposed model	**93.84** ± 0.03	**89.08** ± 0.04	**13.37**	2.40 × ${10}^{-32}$	7.34 × ${10}^{-30}$
Dilated U-Net	94.77 ± 0.02	90.94 ± 0.03	**35.70**	2.90 × ${10}^{-49}$	1.19 × ${10}^{-47}$	Dilated U-Net	92.68 ± 0.05	87.95 ± 0.06	13.60	4.37 × ${10}^{-40}$	3.57 × ${10}^{-34}$
U-Net	94.43 ± 0.02	90.43 ± 0.03	35.98	4.87 × ${10}^{-42}$	1.93 × ${10}^{-40}$	U-Net	91.30 ± 0.06	86.53 ± 0.08	17.91	3.34 × ${10}^{-31}$	1.84 × ${10}^{-27}$
R2U-Net	94.38 ± 0.04	90.30 ± 0.05	39.19	1.59 × ${10}^{-54}$	4.59 × ${10}^{-53}$	R2U-Net	80.51 ± 0.03	71.60 ± 0.03	19.80	6.98 × ${10}^{-18}$	1.34 × ${10}^{-25}$
Attention U-Net	94.76 ± 0.02	90.94 ± 0.03	38.56	4.90 × ${10}^{-50}$	3.84 × ${10}^{-48}$	Attention U-Net	93.03 ± 0.06	87.79 ± 0.09	15.26	4.87 × ${10}^{-29}$	8.44 × ${10}^{-26}$
MFP U-Net	94.20 ± 0.02	90.02 ± 0.03	38.28	9.61 × ${10}^{-53}$	8.16 × ${10}^{-51}$	MFP U-Net	93.73 ± 0.04	88.71 ± 0.06	13.40	8.97 × ${10}^{-29}$	4.00 × ${10}^{-27}$

Significant values are given in bold.

**Figure 6:**
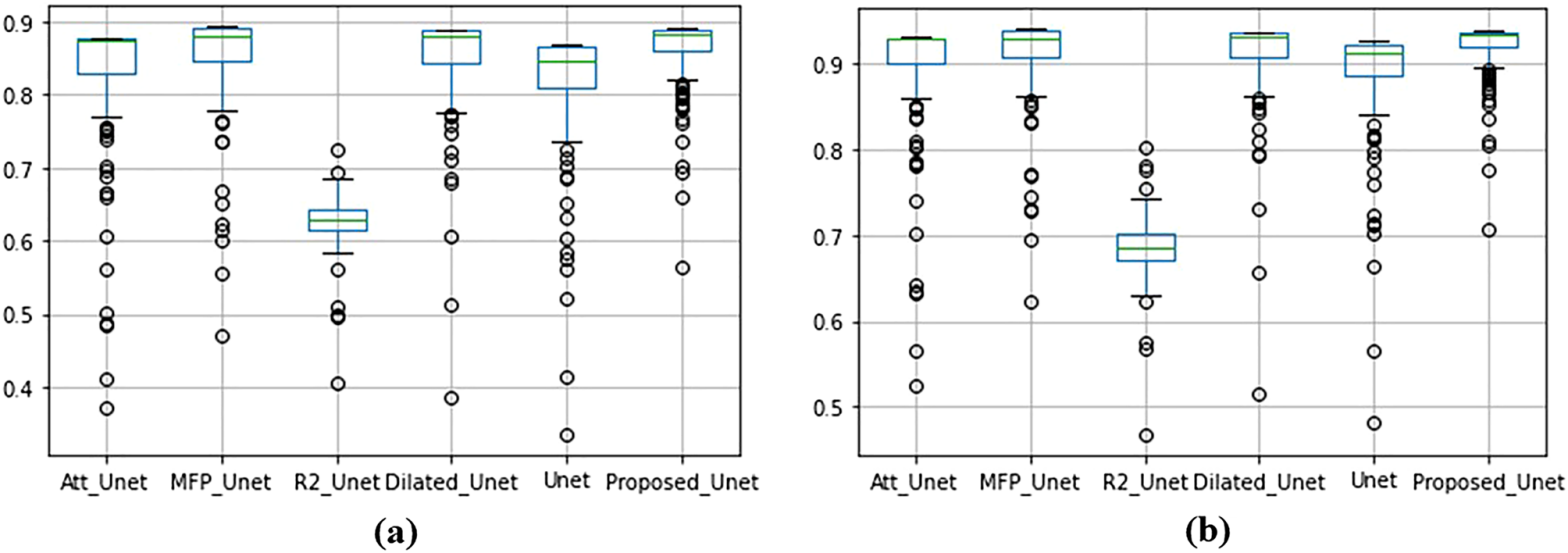
Comparing standard deviations and median results of Dice and Jaccard coefficients for foot data. Dice (**a**), Jaccard (**b**).

Considering the standard deviations, it is confirmed that the results of the proposed method are significant (CRL Dice p-value < 8.80 × ${10}^{-59}$, Foot Dice p-value < 2.40 × ${10}^{-32}$). From the statistical aspect, we know that there are a few cases in which other methods outperformed the proposed method. Moreover, p-value for each method has been computed during training phase. The significant small p-value in all methods indicates that the differences between metrics’ means are remarkable. For more comparison details, a whisker plot was created which comparing all results in Fig. 6 for the foot data. In this figure, the outliers are shown as dots and the green line shows the median of Dice and Jaccard coefficients. The higher dots density that are close to median line as well as outliers dots indicate amount of segmentation accuracy. The figure illustrated that in the proposed model there is no outlier point and all dots are densely nearby median line that indicate better performance in comparison with other models. In both Dice and Jaccard coefficient images (see “a” and “b” of Fig. 6). However, in R2_Unet case, there is number of outlier dots, with less number of dots with spars dispersal of dots close to median line which all together indicate the low segmentation accuracy in comparison with the proposed model.

**Figure 7:**
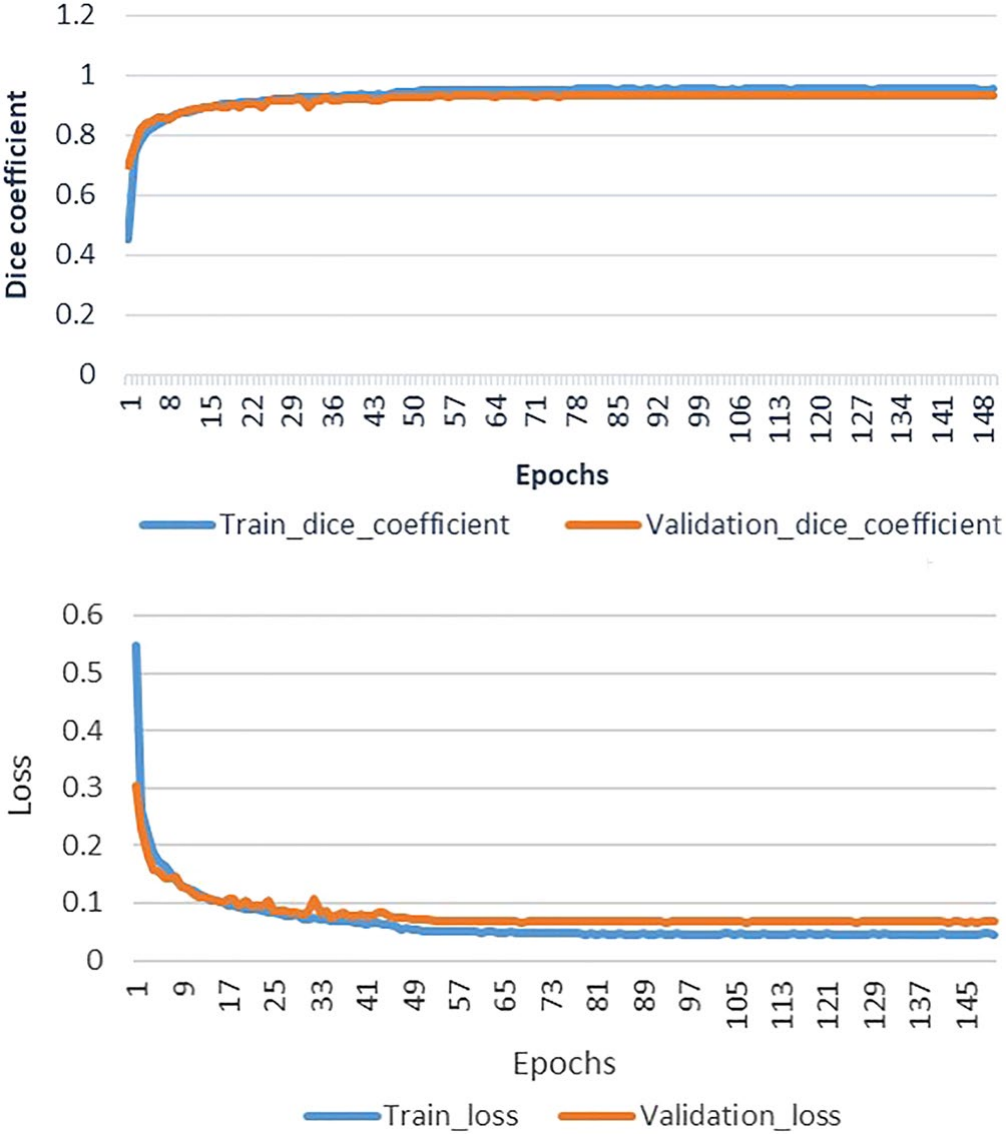
Training and validation dice accuracy and loss plots for the proposed architecture for the CRL segmentation.

Figure 7 illustrates how the losses and accuracy of the proposed model based on the Dice index can change during the training and validation phases for the CRL segmentation. The network converged during the first 15 epochs when the learning rate was fixed to 0.0001 and batch size was 1 during the training phase. However, we found that all the models required around 150 epochs to achieve the best results. As we can see, the loss decreased and dice accuracy increased exponentially in the first 15 epochs. The Dice loss function are commonly employed for the class imbalanced datasets, which is common in the medicine domain. In this light, we have used Dice metric as loss function in the proposed model. According to the implementation of Dice loss function, the loss is minus of calculated value of dice coefficient. Either “1-Dice coefficients” or “−Dice coefficients” should make no difference for convergence but just a different way for monitoring since the values are in the range of [0, 1], or [− 1, 0]. Thus, the negative loss values in Fig. 7, is due to minus Dice coefficients (−Dice coefficients) that we have used in this research. However, after re-training the model Fig. 7 in conventional format was added as follows.

**Figure 8:**
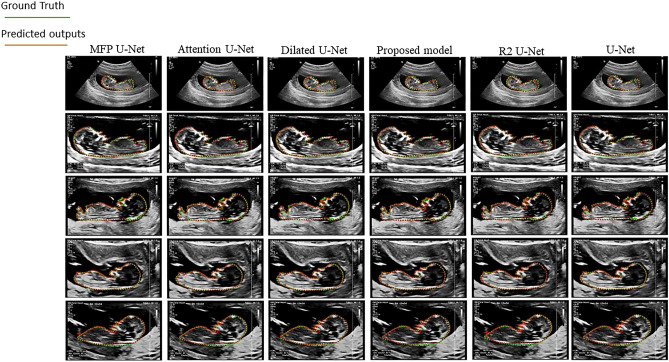
Samples of CRL segmentation achieved by the proposed model in comparison with other U-Net-based models.

**Figure 9:**
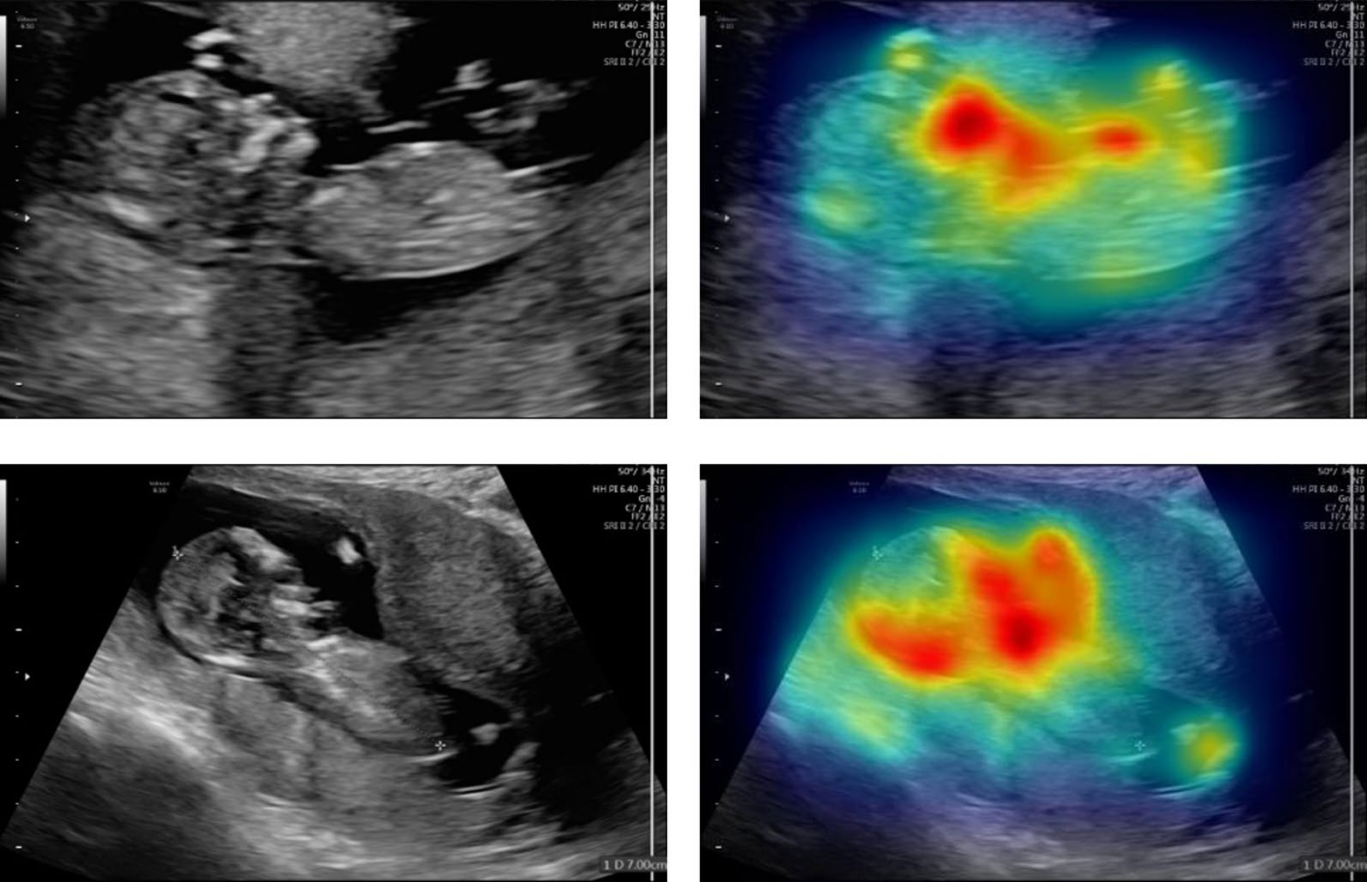
Samples of the salient output results of Net1 for the corresponding image using CRL images.

In Fig. 8, representative samples of the segmented CRL have been shown to compare the results of the proposed model with other U-Net-based architectures. In these figures, the ground truth and the predicted contours are indicated in green and red, respectively. After the visual inspection, we concluded that all methods have very competitive performance; however, quantitative metrics demonstrated that the proposed method outperformed even the best performing Unet-based architectures with 1.02% and 0.68% improvement in DSC and Jaccard indices for the CRL segmentation, respectively. In order to demonstrate that Net1 gives the salient effects of the input image, the outcome of the Net1 is displayed in Fig. 9.

**Figure 10:**
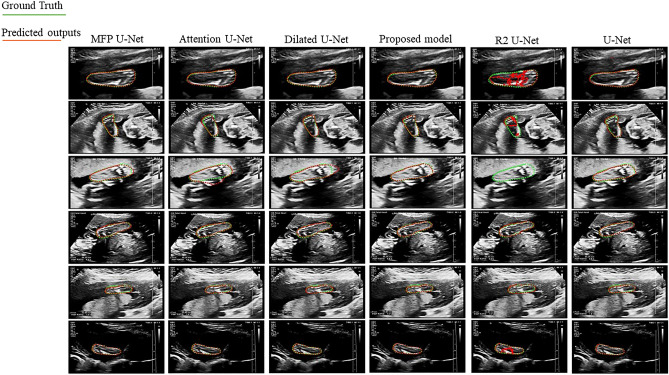
Representative results of fetal foot segmentation achieved by the proposed model in comparison with other U-Net based models.

**Figure 11:**
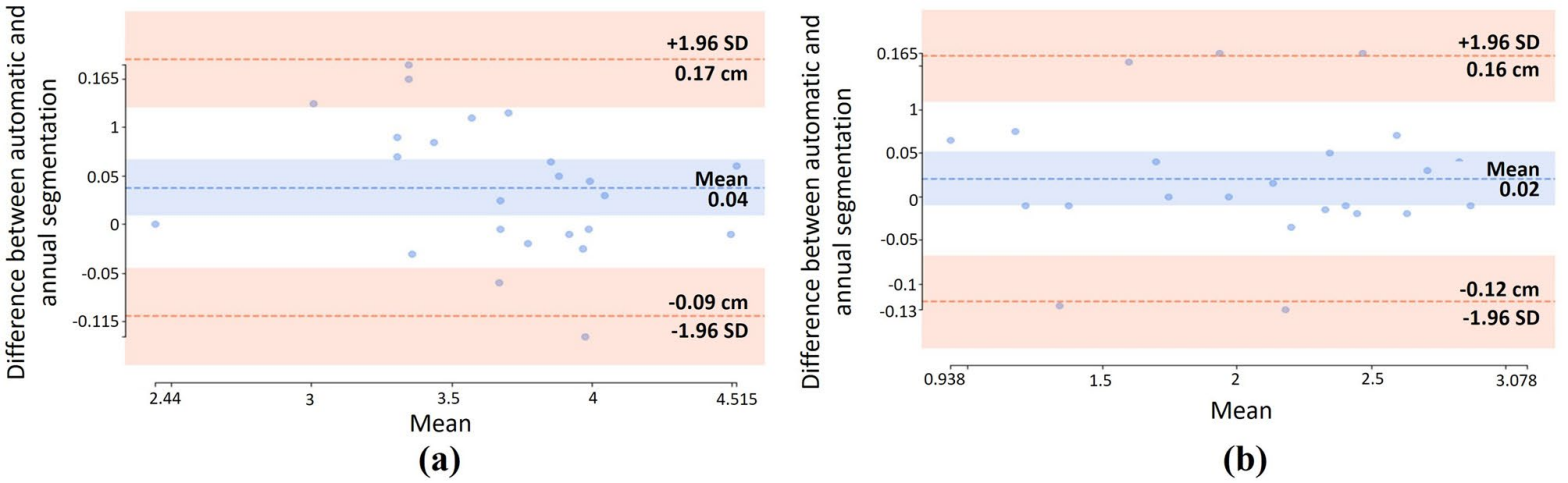
Bland–Altman for CRL and fetal foot length measurement in test set. CRL **(a)**, fetal foot **(b)**.

Furthermore, we compared the performance of the proposed network for fetal foot segmentation in ultrasound images. Similar results were observed and representative samples are shown in Fig. 10 (references are indicated in green and predicted contours in red). The experimental results obtained from the state-of-the-arts U-Net-based segmentation networks are reported in Table 2. Compared to the other architectures, we observed that through using the proposed architecture, performance improved on average between 2–3% in terms of DSC. Using the proposed model, the average DSC and Jaccard of 93.84% and 89.08% were also obtained for the fetal foot segmentation, respectively. The length of the CRL and fetal foot has measured throughout the automatic and manual segmentation manner. The correlation and Bland–Altman analyses^53^ has computed using the results of previous mentioned measurements. Figure 11 illustrates the Bland–Altman graphs of the differences, using the random selected samples of test dataset for length measurement of the segmented parts in CRL and foot data.

### Experiments on CVC-ClinicDB dataset

In order to show the effectiveness of the proposed architecture, in comparison with the other U-Net-based architectures, the CVC-ClinicDB dataset was evaluated. It was revealed that the low contrast of the structures in the CVC-ClinicDB dataset makes the identification of the polyp more challenging. Table 3 reports the DSC and JSC values obtained from different models. The proposed model exhibited superior performance compared to the other U-Net models. Interestingly, these results show an improvement of 6.39%, and 8.85% in DSC, and JSC indices, respectively, compared to the best performing U-Net model (dilated U-Net), which confirms the effectiveness of the proposed model in a more challenging dataset. By considering that the CVC-ClinicDB dataset is a public dataset used for polyp segmentation, we have compared the proposed algorithms with the existing works and with the result of some base approaches^32^, and U-Net based approaches like U-Net^4^, PraNet^15^, and Res U-Net++^54^. Table 4 reports the DSC of the proposed method and compare with other mentioned approaches in this case. On CVC-ClinicDB, our model achieves a dice coefficient of 83%. From the results in Table 4, we concluded that, compared to traditional approaches, the proposed method achieved much better results on DSC. However, most of the U-Net based models cannot yield outstanding results on CVC-Clinic-DB dataset at the same time in comparison with the proposed method. But, some U-Net based methods like PraNet^15^, indicates improvement and outperformed the proposed method throughout the CVC-ClinicDB dataset (83% of proposed method in comparison with 89.90% of PraNet). It is noticeable that the test condition in this paper and in^15^ is not similar where test data selected in a random manner and test samples are not similar. Moreover, 20% and 10% of the CVC-ClinicDB dataset randomly selected as test data in this paper and in^15^, respectively.

**Table 3:** Experiment results on CVC-Clinic public dataset for polyp segmentation using proposed and other U-Net based models.

Model	Proposed model	MFP U-Net	R2U-Net	Dilated U-Net	Attention U-Net
DSC	**83.00**	66.80	55.05	76.61	39.74
JSC	**75.31**	58.58	48.09	66.46	31.12

Significant values are given in bold.

**Table 4:** Comparison the results of the proposed model with state-of-the-art results on CVC-ClinicDB dataset.

Methods	DSC
Proposed method	83.00
Guo et al.^32^	69.69
Sun et al.^55^	82.84
Banik et al.^56^	81.30
Ronneberger et al.^4^	64.19
Fan et al.^15^	89.9
Zhou et al.^57^	79.4
Jha et al.^54^	79.55

**Figure 12:**
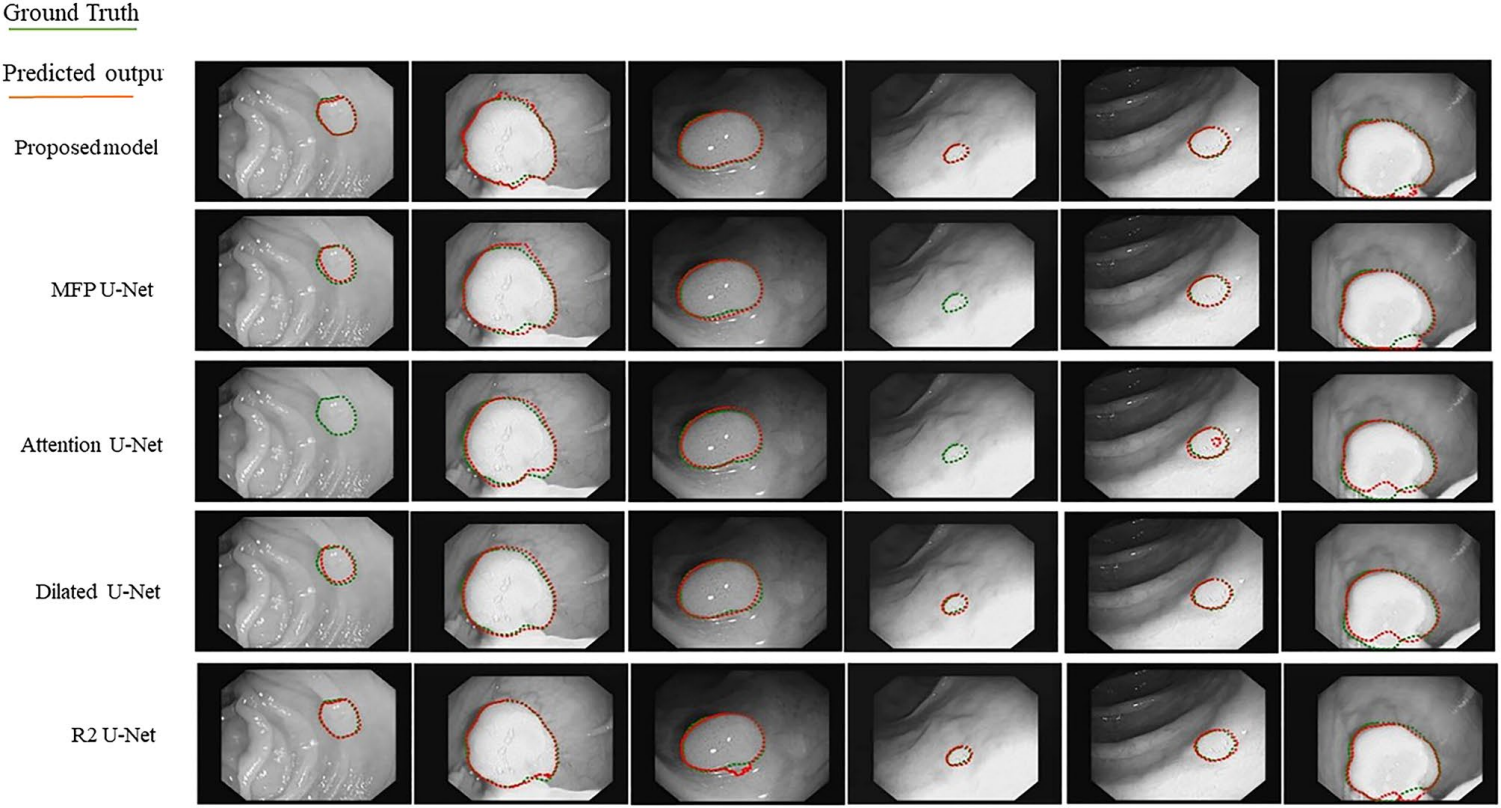
Representative results of segmentation achieved by the proposed model in comparison with other U-Net based models on CVC-ClinicDB dataset.

Figure 12 depicts the segmentation results obtained from different models on CVC-ClinicDB dataset. In these figures, green and red contours show the ground truth and predicted labels, respectively. The overall quantitative analysis showed that the proposed model performed efficiently in a more challenging dataset with flat and small polyps (such as the first and fourth columns).

### Experiments on multi-site MRI dataset

For more evaluation and to show the robustness of the proposed model in comparison with different medical image segmentation models, the proposed model was validated on multi-site MRI dataset for prostate segmentation. The quantitative results of the proposed approach and other U-Net base models are presented in Table 5. Table 5 represented the results on multi-site MRI dataset throughout the three separated sites D, E, F (There are new and robust researches on these three sites of the mentioned dataset for comparison). We have used 171, 243, and 121 images from sites D, E, AND F respectively in our experiments. From Table 5, It is observed that our model has achieved more accurate and stable segmentation results. We evaluated each site separately and compared with state-of-the-art results (Table 5). The proposed model outperformed other methods with 91.55%, 90.85%, and 90.75% for sites D, E, and F, respectively.

**Table 5:** Comparison the Dice coefficients result of the proposed model with other U-Net models as well as state-of-the-arts on multi-site MRI dataset separately.

Approaches	Site D	Site E	Site F
**Proposed method**	**91.55**	**90.85**	**90.75**
MFP U-Net^35^	84.89	82.58	85.53
Attention U-Net^26^	87.63	88.64	88.85
Dilated U-Net^36^	89.22	88.84	89.41
R2U-Net^25^	59.26	65.36	81.19
U-Net^4^	85.43	90.62	86.15
JiGen^58^	86.00	86.00	88.00
BigAug^59^	87.66	81.20	88.96
Epi-FCR^60^	86.55	80.63	89.76
RSC^61^	86.21	79.97	89.80
FedAvg^62^	86.30	80.38	89.15
ELCFS^63^	88.23	83.02	90.47

Significant values are given in bold.

**Figure 13:**
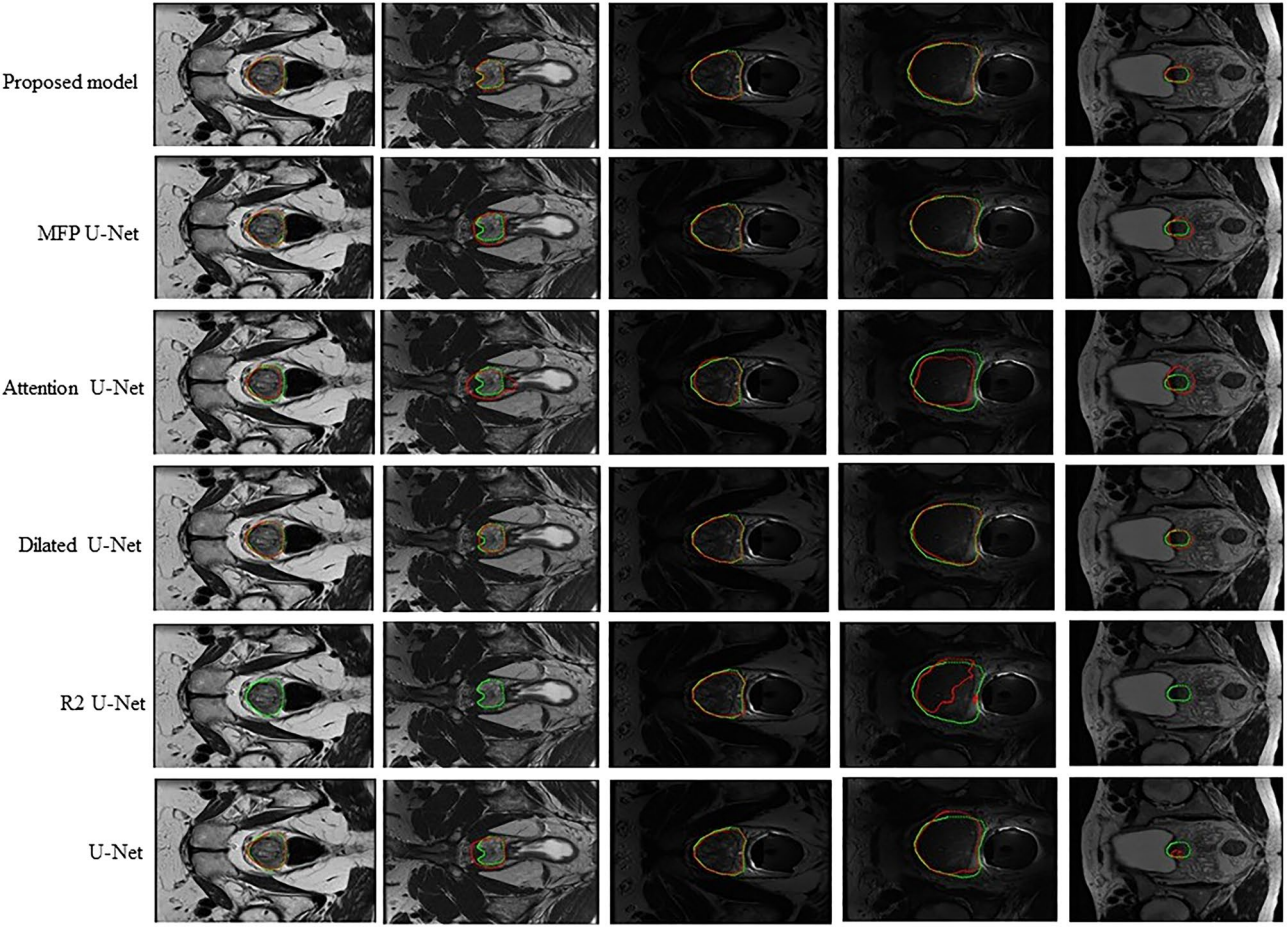
Representative results of segmentation achieved by the proposed model in comparison with other U-Net based models on multi-state MRI dataset.

From Table 5, it is concluded that the proposed model has accurate and also stable segmentation result. It is noticeable that site D includes more samples than site F, but in site D all methods resulted in worse segmentation accuracy in comparison with results on site F, because of more challenging data. But the proposed model illustrated stable result even on challenging data. Figure 13 shows our proposed method in comparison with other U-Net based approaches for segmentation of prostate in some random selected MRI images by considering their corresponding ground truth set (green line). Results illustrated that compared with the other U-Net models, the proposed model produces more accurate segmentation mask and delineates the clear boundary for MRI data. The worst segmentation performance is related to R2U-Net model that could not segmented in three samples (samples in column 1, 2, and 5). Results demonstrated that the proposed model has well performance in face of challenging MRI data (small prostate in column 5 of Fig. 12), while other models did not show promising performance in face of such challenging data.

## Discussion

In this paper, we have proposed a novel U-Net based model known as Double Attention Res-U-Net which was applied for CRL, fetal foot, polyp, and prostate segmentation in different types of clinical images. The proposed model includes two novel consecutive residual U-Net based architectures. Using Attention Gates (AGs) in different scales along with residual blocks in two subsequent U-Net networks encourage the model to generate richer contextual information to abstract using the networks. This model improved the segmentation accuracy in all clinical target segmentation throughout the different image types due to the above-mentioned structures.

The architecture of the proposed model includes two distinct consecutive networks. Each of them consists of four main blocks including encoding blocks, decoding blocks, residual blocks, and AG blocks. Residual blocks (Fig. 2) are located between the encoder and the decoder paths in both networks. The AGs (Fig. 3) have been used within the skip connections of both networks in order to enable the networks to concentrate on key features with more effective performance in segmentation procedure. Moreover, we have used the AGs in different scales in order to encourage the models to extract richer features with different resolutions. This scheme is likely to increase the effectiveness of the extracted feature maps for the segmentation process. It is noticeable that the input of the second network (Fig. 1) is an element-wise multiplication of the output and input of the first network. In addition, the proposed model benefits from the simple encoder and decoder architecture in both networks; itis comprised of a 2D-convolutional layer, a batch normalization layer, and a rectified linear unit as an activation function. This simple and tuned architecture in the encoder and decoder paths leads to simple computation for extracting well-suited features without extraordinary computations.

To properly evaluate the performance of this proposed method, three different datasets were included in this paper. The qualitative and quantitative assessment using three different types of clinical images (i.e., the collected ultrasound dataset for CRL and fetal foot segmentation purpose as well as the CVC-ClinicDB dataset for polyp segmentation task as well as multi-site MRI dataset for prostate segmentation task) proved that the proposed architecture improved the segmentation performance in comparison with the state-of-the-art U-Net based models, recently being investigated for the medical image segmentation task. Results of the present study illustrated that the proposed architecture generally produces more precise results than dilated U-Net, U-Net, R2Unet, attention U-Net, and MFP U-Net (Tables 2, 3, and 4). This superiority results from the richer contextual feature maps extracted while using attention gates in different scales along with residual blocks in the two subsequent well-structured and simple U-Net networks. The proposed architecture achieved Dice and Jaccard coefficients of 95.79%, 91.62% respectively for CRL, and 93.84%, 89.08% for fetal foot, and 83%, 75.31% for polyp segmentation, and 92.07%, 87.14% for prostate segmentation. Regarding Tables 2, 3, 5 our approach led to promising results in comparison with other approaches, while the poorest results were observed in MFP U-Net for CRL, R2Unet in fetal foot, and attention U-Net for polyp segmentation task, and R2U-Net for prostate segmentation among other U-Net based models. The statistical analysis of different approaches indicated that other competitive approaches will show different performance in face of different input data types, but the proposed architecture outperformed the other models in all cases and enjoyed benefits of stability.

The visualized results in Figs. 8, 10, 12, and 13 showed that the proposed approach shows the most agreement with the ground truth segmentation (the green area indicates the ground truth label and the red area shows the predicted label). After the visual analysis, we concluded that more models have competitive performance, but R2U-Net demonstrated the worst performance in face of challenging foot data, while MFP U-Net and attention U-Net did not show promising performance in face of challenging polyp data (flat and small polyp) like the first and the fourth columns in Fig. 12. From Figs. 8, 10, 12, and 13, we observed that the proposed model has stability in performance even in the face of challenging data and outperformed the other models. For instance, in multi-site MRI dataset, site D contains more samples than site F, but due to more challenging data in site D, all methods resulted in worse segmentation accuracy in site D in comparison with results on site F, while the proposed model illustrated stable result even on site D. Moreover, the statistical analysis was performed and the standard deviation as well as the p-values were computed (Table 2) for all approaches. The significant small p-value in the proposed method indicates that the differences between metrics’ means are much significant in comparison with other more related approaches.

## Conclusion

In this work, we introduced a novel U-Net-based model known as Double Attention Res-U-Net for the purpose of different clinical target segmentation in different types of medical images (crown rump length, and fetal foot segmentation in ultrasound imaging system, polyp identification in colonoscopy images, as well as prostate segmentation in MRI images). The presented system automatically measured the fetal foot and CRL from images of fetal body, segmented the polyp in colposcopy images, and segmented prostate targets from MRI data. Compared with the other U-Net-based architectures, the proposed model consists of two networks that are composed of encoder-decoder modules with five tuned blocks for encoding and decoding the data. Each network is comprised of a modified residual structure to produce more high-level features and retain more spatial features between encoding and decoding modules. To focus on the most relevant information at different scales/resolutions, attention gates were employed. To validate our approach, three different segmentation datasets were used for the task of CRL, fetal foot segmentation from ultrasound images, polyp segmentation from colonoscopy imaging system, and prostate targets from MRI images. Quantitative analysis showed superior performance of the proposed model in comparison with the state-of-the-art U-Net-based models in all data types. Moreover, the proposed architecture indicated significant improvement accuracy for polyp segmentation in comparison with other U-Net based models and achieved 83% and 75.31% in Dice and Jaccard coefficients respectively. These results show an improvement of 6.39%, and 8.85% in Dice, and Jaccard indices, respectively, compared to the best performing U-Net model (dilated U-Net), which confirms the effectiveness of the proposed model in a more challenging dataset. Results of prostate data demonstrated that the proposed model has well performance in face of challenging MRI data (small prostate), while other models did not show well performance in face of such challenging data.

## Data Availability

The fetal CRL datasets analyzed during the current study are available in the CRL repository, https://figshare.com/articles/dataset/CRL/16570518. The fetal foot datasets analyzed during the current study are available in the foot repository, https://figshare.com/articles/dataset/Foot/16570566. The colonoscopy datasets analyzed during the current study are available in the CVC-ClinicDB repository, https://www.dropbox.com/s/p5qe9eotetjnbmq/CVC-ClinicDB.rar?dl=0. The MRI datasets analyzed during the current study are available in the Multi-site Dataset for Prostate MRI Segmentation repository, https://liuquande.github.io/SAML/. We confirm that all methods were carried out in accordance with relevant guidelines and regulations.
